# Association between the Range of Motion and Passive Property of the Gastrocnemius Muscle–Tendon Unit in Older Population

**DOI:** 10.3390/healthcare9030314

**Published:** 2021-03-12

**Authors:** Masatoshi Nakamura, Shigeru Sato, Ryosuke Kiyono, Kaoru Yahata, Riku Yoshida, Taizan Fukaya, Satoru Nishishita, Andreas Konrad

**Affiliations:** 1 Institute for Human Movement and Medical Sciences, Niigata University of Health and Welfare, 1398 Shimami-cho, Kita-ku, Niigata City, Niigata 950-3198, Japan; masatoshi-nakamura@nuhw.ac.jp (M.N.); hpm19006@nuhw.ac.jp (S.S.); hpm19005@nuhw.ac.jp (R.K.); hpm20011@nuhw.ac.jp (K.Y.); fukaya.taizan@gmail.com (T.F.); 2 Department of Physical Therapy, Niigata University of Health and Welfare, 1398 Shimami-cho, Kita-ku, Niigata City, Niigata 950-3198, Japan; hpa17123@nuhw.ac.jp; 3 Department of Rehabilitation, Kyoto Kujo Hospital, 10 Karahashirajoumoncho, Minami-ku, Kyoto 601-8453, Japan; 4 Institute of Rehabilitation Science, Tokuyukai Medical Corporation, Osaka 560-0054, Japan; satoru@rehalab.jpn.org; 5 Kansai Rehabilitation Hospital, Tokuyukai Medical Corporation, Osaka 560-0054, Japan; 6 Institute of Human Movement Science, Sport and Health, University of Graz, Mozartgasse 14, A-8010 Graz, Austria

**Keywords:** rate of force development, shear elastic modulus, plantar flexor, ultrasound

## Abstract

Range of motion has been widely known to decrease with age; however, factors associated with its decrease in the elderly population and especially its gender difference have been unclear. Therefore, this study aimed to investigate the factors associated with ankle dorsiflexion range of motion in the older population. Both male (n = 17, mean ± SD; 70.5 ± 4.2 years; 165.4 ± 5.3 cm; 63.8 ± 7.7 kg) and female (n = 25, 74.0 ± 4.0 years; 151.2 ± 4.9 cm; 50.1 ± 5.6 kg) community-dwelling older adults participated in this study. The ankle dorsiflexion and passive torque of both legs were measured using a dynamometer, and shear elastic modulus of the medial gastrocnemius muscle at 0° ankle angle was measured using ultrasonic shear wave elastography. In this study, we defined the passive torque at dorsiflexion range of motion (DF ROM) as the index of stretch tolerance, and shear elastic modulus as the index of passive muscle stiffness. The partial correlation coefficient adjusted by age, height, weight, and side (dominant or nondominant side) was used to analyze the relationship between DF ROM and passive torque at DF ROM or shear elastic modulus of MG in each male and female participant, respectively. Our results revealed that dorsiflexion range of motion was significantly associated with passive torque at dorsiflexion range of motion in both male (r = 0.455, *p* = 0.012) and female (r = 0.486, *p* < 0.01), but not with shear elastic modulus in both male (r = −0.123, *p* = 0.519) and female (r = 0.019, *p* = 0.898). Our results suggested that the ankle dorsiflexion range of motion could be related to the stretch tolerance, but not to passive muscle stiffness in community-dwelling elderly population regardless of gender.

## 1. Introduction

Generally, range of motion (ROM) has been known to decrease with age [1,2,3,4], with previous studies showing that decreased ROM could lead to declined locomotion and balance [5,6]. In addition, given that declined ROM could lead to an increased risk for falls [7], maintaining and improving ROM among elderly individuals is assumed to be imperative.

Passive muscle stiffness has been thought to be a factor related to ROM. However, Magnusson et al. investigated the individual variability of ROM among young participants and reported that those with greater ROM (flexibility) could tolerate a greater passive torque at the end ROM than those with smaller ROM [8]. These results suggested that individual variability in ROM is related to that in sensation to pain and/or muscle stretch (commonly known as stretch tolerance) rather than elasticity parameters in the muscle (e.g., stiffness).

Recently, the shear wave elastography (SWE) function has been used to measure muscle stiffness. Using this method, Miyamoto et al. (2018) have investigated the factors related to ankle dorsiflexion (DF) ROM in young men and women [9]. These results showed that ankle DF ROM was related to both passive muscle stiffness and stretch tolerance in young men. However, in women it was related to stretch tolerance only. In addition, the knee extension ROM has been reported to be moderately correlated with passive muscle stiffness of the hamstrings of young men [8]. These results showed the possible gender difference of individual ROM variabilities and stretch tolerance could have more influence on the ROM than passive muscle stiffness, especially in young women.

In addition to a study of young adults, Hirata et al. investigated factors related to ROM in community-dwelling male elderly individuals and showed that that stretch tolerance, but not passive muscle stiffness, was associated with ankle DF ROM [4]. However, elderly women and men should be included in the further study as a previous study showed gender differences among factors related to ankle ROM in young adults [10]. Therefore, this study aimed to investigate the association between DF ROM and stretch tolerance or passive muscle stiffness in both community-dwelling elderly men and women. As mentioned above, ROM decreased with aging leads to decreased mobility and balance function and increased risk of falling. Information on the future treatment method should be established to elucidate factors associated with age-related decreased ROM.

## 2. Materials and Methods

### 2.1. Participants

In total, 42 older adults (17 elderly men and 25 elderly women) participated in this study, and their baseline characteristics (mean ± standard deviation (SD)) were an age of 70.7 ± 4.1 years; height of 160.6 ± 8.0 cm; and body mass of 58.7 ± 9.5 kg in males, and 74.0 ± 4.0 years; 151.2 ± 4.9 cm; and 50.1 ± 5.6 kg in females. The experiments were conducted in the movement laboratory at the Niigata University of Health and Welfare.

We measured the DF ROM, passive toque during passive stretching test and shear elastic modulus used by shear wave elastography in both legs in random order to investigate the association between DF ROM and passive torque at DF ROM (i.e., stretch tolerance) or shear modulus in each older males and females. The inclusion criteria in this study were an age of >65 years, residing in the community, and able to walk independently (with or without a cane). The exclusion criteria were cognitive impairment, severe cardiac or musculoskeletal disorders, previous pulmonary disease diagnosis, and hearing impairment. Almost all participants had a sedentary lifestyle, but some participants (men: 29.4%, and women: 32%) were engaging in regular exercise at the time of the experiment. All participants were fully informed of the study procedures and purpose, and all provided written informed consent. This study was approved by the ethics committee at the Niigata University of Health and Welfare, Niigata, Japan (#18104), and conducted in accordance with the Declaration of Helsinki.

The sample size required for a correlation analysis (effect size = 0.5 (large), α error = 0.05, and power = 0.80) was calculated using the G* power 3.1 software (Heinrich Heine University, Düsseldorf, Germany) based on a previous study [9], and more than 26 participants were required in this study. Thus, in this study, we adopted 34 legs for elderly men and 50 legs for elderly women.

### 2.2. Assessment of Dorsiflexion Angle and Passive Torque during Passive Stretching

**Figure 1:**
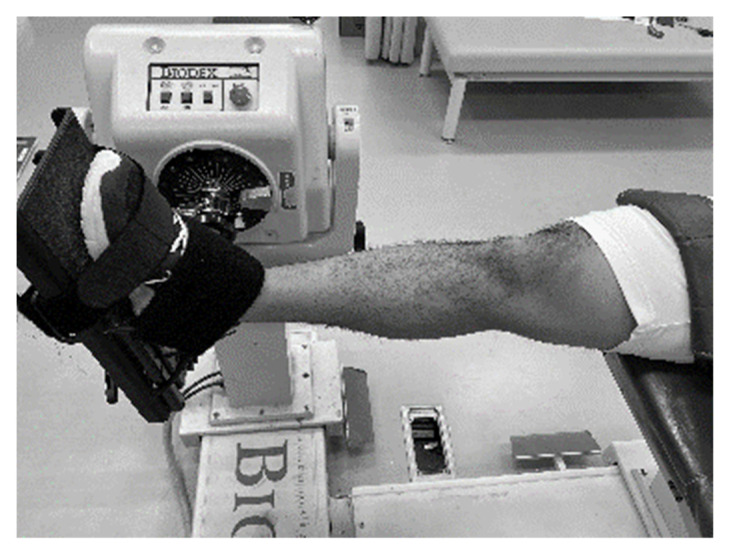
The experimental set-up for range of motion and passive torque measurement.

Each participant was instructed to sit on a dynamometer chair (Biodex System 3.0, Biodex Medical Systems Inc., Shirley, NY, USA) with the hip flexion angle at 70° and 0° knee angle (i.e., anatomical position). In addition, the thigh, pelvis, and trunk of a participant were fixed with Velcro straps strictly (Figure 1). Based on previous studies [11,12,13], the ankle joint was fixed for the footplate of the dynamometer, and the footplate was passively and isokinetically dorsiflexed at angular speed 5°/s from 0° of ankle position. The measurements were performed until the participants felt discomfort, stopping the dynamometer by activating the safety trigger [11,12,13]. We defined the angle and passive torque of this angle as DF ROM and passive torque at DF ROM. Based on previous studies [14,15,16], the passive torque at DF ROM was defined as the sensor for stretching perception, e.g., stretch tolerance. In this study, two warm-up cycles were performed before measurement, avoiding a conditioning effect of the passive dorsiflexion test on the passive stiffness of the muscle–tendon unit [17,18]. After the two warm-up cycles, DF ROM and passive torque were measured twice, utilizing the averages for further analysis. Throughout the DF ROM and passive torque measurement, all participants were instructed to relax and not to offer any voluntary muscle contraction. In addition, we confirmed the absence of voluntary muscle contraction by checking the passive torque-angle curve. In this study, both legs were measured in a random order.

### 2.3. Assessment of the Shear Elastic Modulus

We measured the shear elastic modulus of the medial gastrocnemius muscle (MG) via ultrasonic SWE (Aplio 500, Toshiba Medical Systems, Tochigi, Japan) using a 5–14-MHz linear probe. The measurement position is similar to the DF ROM and passive torque measurement position. The measurement site for shear elastic modulus of MG was 30% of the lower leg length, which was defined from the popliteal crease to the lateral malleolus [11,19]. We obtained the elastographic images in the long-axis MG image at 0° of ankle position (neutral position), and we analyzed the image used with a custom-made image analysis software (MSI Analyzer version 5.0, Rehabilitation Science Research Institute, Osaka, Japan). We set the quadrangular region of interest (ROI) as large as possible within the color-coded area of MG, and we accounted for the artifact from the aponeurosis. Based on the previous studies [13,20], we calculated the shear elastic modulus by dividing the obtained Young’s modulus in the ROI by 3.

### 2.4. Statistical Analysis

Descriptive data are presented as mean ± standard deviations. We used SPSS version 24.0 (IBM Corp., Armonk, NY, USA) for statistical analyses. The partial correlation coefficient adjusted by age, height, weight, and side (dominant or nondominant side) was used to analyze the relationship between DF ROM and passive torque at DF ROM or shear elastic modulus of MG in each male and female participant, respectively. The significance threshold was set to α = 0.05.

## 3. Results

**Figure 2:**
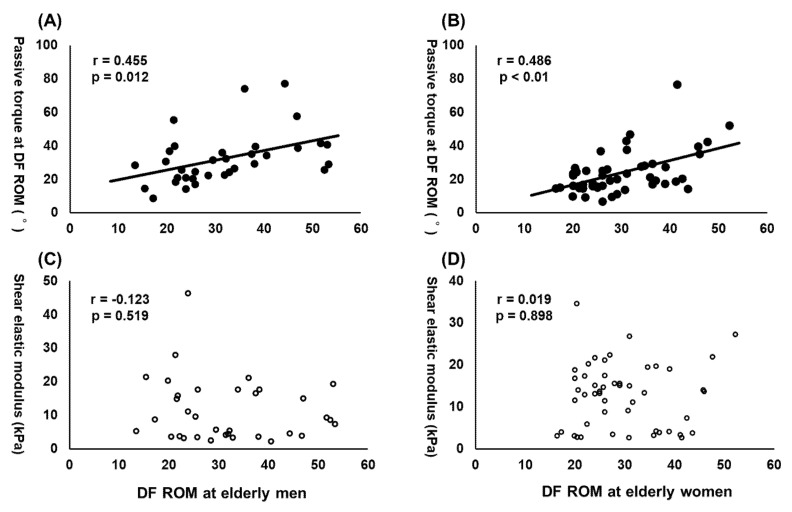
The association between dorsiflexion range of motion (DF ROM) and passive torque at DF ROM (**A**: elderly men, **B**: elderly women) or shear elastic modulus (**C**: elderly men, **D**: elderly women)**.**

The outcome variable and results of partial correlation coefficients are shown in Table 1 and Figure 2. With age, height, weight, and side (dominant or nondominant side) as control variables, DF ROM was significantly associated with passive torque at DF ROM in both male (r = 0.455, *p* = 0.012) and female (r = 0.486, *p* < 0.01), whereas DF ROM was not significantly associated with shear elastic modulus of MG in both male (r = −0.123, *p* = 0.519) and female (r = 0.019, *p* = 0.898).

## 4. Discussion

This study investigated factors related to the ankle DF ROM in community-dwelling elderly men and women. Our results showed that DF ROM was related to passive torque at DF ROM but not passive muscle stiffness. Hirata et al. (2020) reported that the ankle DF ROM in elderly men was related to stretch tolerance, not to passive muscle stiffness of MG [4]. To the best of our knowledge, this is the first study showing that DF ROM was significantly associated with stretch tolerance, but not with passive muscle stiffness in not only elderly men but also women.

In this study, DF ROM was not significantly associated with passive shear modulus. Miyamoto et al. investigated factors related with ankle DF ROM in young men and women, and showing that DF ROM was significantly associated with passive muscle stiffness in only young men but not in women [9]. Moreover, Hirata et al. investigated the association between young and elderly men, showing that DF ROM was significantly associated with passive muscle stiffness in only young men but not in elderly men [4]. Results of this study were consistent with these previous studies, and considering these studies, the ankle DF ROM could be related to passive muscle stiffness in young men; however, the association was weakened with age in both men and women. A previous study showed that the intramuscular perimysium and endomysium contents increase with age [21] and passive muscle stiffness is considerably affected by the connective tissue within the muscle (e.g., perimysium and endomysium) [22]. Therefore, it is assumed that passive muscle stiffness is increased with age. However, Nakamura et al. (2017) reported that DF ROM in elderly women was lower than that in young women, but there was no significant difference of shear elastic modulus of MG between elderly and young women [23]. Since there is no significant age-related change in passive muscle stiffness of MG, differences in ROM might be rather associated by stretch tolerance than by passive muscle stiffness [4,9].

Conversely, results of this study revealed that DF ROM was significantly associated with age-adjusted passive torque in both males (r = 0.455, *p* = 0.012) and females (r = 0.486, *p* < 0.01). As previous studies reported that the passive torque at DF ROM is an indicator of stretch tolerance [14,15,16], our results showed that DF ROM in older adults including both men and women was related with stretch tolerance, but not with passive muscle stiffness. Our results supported and extended results of a previous study by Miyamoto et al. (2018) [9] investigating young men and women [9], and that by Hirata et al., investigating young and older men [4]. To date, factors related with stretch tolerance have been unclear; however, our results suggested that the association between DF ROM and passive muscle stiffness becomes weaker with age, whereas the association between DF ROM and stretch tolerance persists.

As described above, decreased ROM could lead to declined locomotion and balance [5,6] and increased risk for falls [7], maintaining and improving the ROM among elderly individuals. Previous studies showed that the ROM was improved after the stretching intervention in older adults [3,23]. Conversely, since our results showed that DF ROM was significantly associated with stretch tolerance, but not with passive muscle stiffness in elderly individuals, the intervention method could more effectively change the stretch tolerance among older adults. A previous study showed that both static stretching and hold–relaxation stretching could change the stretch tolerance and increase the ROM in elderly individuals [24]. Therefore, both static and hold–relaxation stretching could be an effective strategy to improve the ROM in elderly adults due to stretch tolerance changes. Consequently, this could on the one hand lead to improvements in locomotion and balance and on the other hand to a decrease in risk of falls.

## 5. Conclusions

Our results showed that DF ROM was not significantly associated with shear elastic modulus of MG, but significantly associated with passive torque at DF ROM in both the male and female older population. This suggests that stretch tolerance is an important factor for ROM in elderly individuals.

## Figures and Tables

**Table 1:** The outcome variables in both elderly men and women.

		Mean ± SD	Range
Male participants (17 men) N = 34 legs	Dorsiflexion range of motion (DF ROM) (°)	31.7 ± 11.4	13.4–53.3
	Passive torque at DF ROM (Nm)	32.3 ± 15.1	8.7–77.2
	Shear elastic modulus (kPa)	11.4 ± 9.2	2.2–46.4
Female participants (25 women) N = 50 legs	DF ROM (°)	29.7 ± 8.8	16.4–52.2
	Passive torque at DF ROM (Nm)	27.2 ± 14.3	7.0–76.6
	Shear elastic modulus (kPa)	12.7 ± 7.6	2.6–34.6

## Data Availability

All data generated or analyzed during this study are included in this published article.
